# Childhood Malnutrition: Time for Action

**DOI:** 10.3390/children8020103

**Published:** 2021-02-03

**Authors:** Tonia Vassilakou

**Affiliations:** Department of Public Health Policy, School of Public Health, University of West Attica, Athens University Campus, 196 Alexandras Ave, GR-11521 Athens, Greece; tvasilakou@uniwa.gr

Childhood malnutrition of every form, including undernutrition (wasting, stunting and underweight), micronutrient deficiencies, as well as overweight and obesity, consists a triple burden of disease, especially for low- and middle-income countries, and is one of the leading causes of poor health and a major impediment to personal development and achievement of full human potential worldwide [1]. Globally in 2019, 149 million children under the age of 5 years were stunted, almost 50 million wasted, 340 million suffered from micronutrient deficiencies [2] and 38,2 million were overweight and obese [3]. The nutritional needs of children and adolescents are unique and poor availability or limited access to food of adequate nutritional quality leads large population groups to undernutrition, poor nutritional status, overweight and obesity. These malnutrition forms often exist simultaneously and are interconnected [4].

Malnutrition is a global public health problem that is associated with high health care cost, and increased morbidity and mortality [5]. Approximately 45% of deaths among children under 5 years of age can be attributed to undernutrition [6]. Childhood undernutrition may result in long-term effects that are irreversible, including impaired physical growth and cognitive development [7,8,9]. Furthermore, undernutrition may reduce sensory-motor abilities, reproductive function and increase children’s vulnerability to infections and hereditary diseases, such as diabetes [9,10]. Moreover, undernutrition causes raise of health care costs, reduction in human productivity at adulthood, and shrinkage of economic development, which can result to a long-term cycle of poverty and illness. Childhood undernutrition mostly occurs in low- and middle-income countries, mainly due to poverty, which is associated with suboptimal feeding practices, poor sanitary conditions and insufficient health care services [11,12,13,14,15].

While there has been some progress concerning the reduction of undernourished population from over one billion people in the 1990s to 793 million in 2015 [14], around two billion people suffer from micronutrient deficiencies or “hidden hunger” [16,17]. Regarding the situation among children, globally one-third of them are suffering from micronutrient deficiencies [18]. Hidden hunger poses a major threat to health and development of populations worldwide, particularly among children and pregnant women in low-income countries [11,19]. The health effects of micronutrient deficiency include impaired physical growth, weight loss, immune system vulnerability [19], neurological disorders, cardiovascular diseases, megaloblastic anaemia, and skin problems [18,19,20]. Furthermore, recent research findings on the developmental origins of disease have indicated that both fetal and infant under- and overnutrition are serious risk factors for obesity with adverse consequences throughout the life cycle [21,22].

At the same time, both in high-income and low- and middle-income countries, rates of childhood overweight and obesity are rising [3]. In the past 40 years, the obesity pandemic has changed the existing malnutrition patterns. The prevalence of obesity during childhood and adolescence has risen significantly over the last decades. Since the early 1980s, the prevalence of overweight and obesity increased rapidly, initially in high-income countries [3]. Globally, overweight and obesity prevalence is very high [3,23], especially in Europe [24]. In 2016, obesity was estimated to affect 1,9 billion persons worldwide [3]. The World Health Organization (WHO) has announced that childhood and adolescent obesity is the major public health problem and advises on actions needed to slow the progression of obesity epidemic [25]. According to the National Health and Nutrition Examination Survey (NHANES) study, the rate of obesity among adolescents in the United States has quadrupled during the last decades [26]. The etiology of obesity is multifactorial, including genetic, environmental, such as nutrition and physical activity, and socioeconomic factors [24,27,28,29]. Dietary shifts in recent decades, related to modern lifestyle, higher available income and increased consumption of highly processed foods, combined with low physical activity levels, are considered to contribute to this increase in obesity rates [27,29]. Unhealthy diet is the core problem of the current nutrition situation [3]. Environmental and societal factors, which originate from financial development and absence of substantial supportive policies in infrastructures and services, such as education, health, transport, urban planning, environment, climate change, agriculture, food processing, distribution and marketing, often result in changes of dietary and physical activity patterns [24,27,28,29,30].

The risk of morbidity and mortality in adult life increases among persons who are overweight or obese as children or adolescents [3,26,27,30]. It is well established that obesity and its determinants are risk factors for the main nutrition-related non-communicable diseases (NCDs), including diabetes mellitus [31], cardiovascular diseases (hypertension, coronary heart disease and stroke) [3,32] and certain cancers [3,30]. Unhealthy diet and poor nutritional status are among the most important risk factors for these diseases globally [3].

This Special Issue comprises of both research and review articles, which focus on diverse components of malnutrition among healthy and non-healthy population groups spanning high income and low- and middle-income contexts. Each of the papers provides the readers with a chance to examine a different aspect of childhood malnutrition and highlights the urgent need for design and implementation of the necessary actions and policies for its prevention and control.

Readers are encouraged to explore these articles and consider the role of malnutrition as a risk factor in their own context. Every country in the world and every population group is affected by one or more forms of malnutrition [2,4,29]. Confronting every form of malnutrition is one of the greatest global public health challenges [2,14]. A healthy diet, initiating in the early stages of life, provides adequate energy and nutrient intake, results in healthy weight, and is crucial for the physical, cognitive and mental development of children and adolescents, as well as for their long-term health [3].
